# The Challenge of Vestibular Rehabilitation in a Patient with Bilateral Vestibular Dysfunction Following Surgery: A Case Report

**Published:** 2018-05

**Authors:** Sadegh Jafarzadeh, Mohammad Reza Golrokhian-Sani

**Affiliations:** 1 *Department of Audiology,* * School of Paramedical Sciences, Mashhad University of Medical Sciences, Mashhad, Iran.*; 2 *Department of * *Otolaryngoloy, Sina Hospital, * *Mashhad University of Medical Sciences, Mashhad, Iran.*

**Keywords:** Adaptation, Bilateral vestibular dysfunction, Gentamicin, Ototoxicity, Vestibular rehabilitation

## Abstract

**Introduction:**

Bilateral vestibular dysfunction (BVD) is an uncommon finding in vestibular assessment, and the combination of BVD and orthopedic problems represents a rare and challenging case for treatment.

**Case Report:**

The patient had several previous back surgeries and received gentamycin after surgery. After 6 months, she experienced continuous dizziness, unsteadiness and oscillopsia. The patient underwent complete vestibular assessment and received an individualized vestibular rehabilitation program for 9 months. She achieved a complete recovery from all symptoms and returned to active social function.

**Conclusion:**

Vestibular rehabilitation could be an effective treatment for complicated cases of BVD. Adaptation exercises may be useful in young patients with BVD.

## Introduction

Bilateral vestibular dysfunction (BVD) is an uncommon finding in vestibular assessment (1). In 1989, Macgath found that only 1.6% of 5,499 patients with vestibular disorder had bilaterally abnormal results in a caloric test (2). However, rare conditions such as this have a dramatic effect on a patient's quality of life (3,4), including severe imbalance and oscillopsia (3,5). Some disorders such as bilateral Meniere's disease could lead to BVD, but the main cause of this condition remains ototoxicity (2,6,7). Vestibular rehabilitation could improve the patient's balance and physical function and prevent falling and further disabilities (8). However, there are very few studies in BVD, and those published have predominantly used substitution exercises. These studies show a partial recovery in some of patients (8-11).

## Case Report

The patient in this case was a 16-year-old female with orthopedic problems who had undergone several spine surgeries for kyphoscoliosis, and had received gentamycin after the procedures. After 6 months, the patient experienced continuous dizziness, unsteadiness and oscillopsia after using aminoglycoside (gentamicin). She also had abnormalities in movement due to inner ear and orthopedic problems, especially upon standing and walking. The vertigo was continuous and worsened upon motion, particularly in relation to vertical head movement and walking. Patients with this condition also tend to suffer severe imbalance, oscillopsia, gait abnormality and frequent falling. The patient had been treated by several ear, nose and throat (ENT) specialists and a neurologist with betaserc and cinnarizine since first experiencing vertigo in the 4 months prior to our visit. The patient left school because of her vertigo and imbalance.

Initially, the patient underwent a case history and several auditory tests, and showed normal hearing in pure tone and speech audiometry, good speech discrimination, type A tympanogram and present acoustic reflexes. The result of previous electrocochleography also was normal. The patient did not demonstrate ear fullness, low pitch tinnitus or episodic vertigo. For vestibular assessment, we performed several physical and functional vestibular tests to determine the extent of the abnormality, including electronystagmography (ENG) (Hortmann, Otometrics, Denmark), a Halmagyi head impulse test (HIT), cervical vestibular evoked myogenic potential (cVEMP) (EP25, Intra acoustic, Denmark), ocular vestibular evoked myogenic potential (oVEMP; EP25, Intra acoustic, Denmark), a bedside dynamic visual acuity test (DVT), Romberg test and Persian dizziness handicap inventory (DHI). DHI showed high validity, reliability and internal consistency (12). ENG and HIT are used for assessing semicircular canals; cVEMP and oVEMP evaluate otolith organs; DVT shows visual acuity during movement and is related to vestibular abnormality. The Romberg test is used for balance evaluation, and DHI shows the impact of vertigo and imbalance on the patient.

The result of ENG showed normal ocular motor function, no spontaneous or positional nystagmus and BVD in a caloric test (less than 3° nystagmus for cold and warm stimulus in both ears). The bedside HIT was abnormal, and overt catch-up saccade was observed. The cVEMP and oVEMP results were abnormal on both sides. The patient could stand in the open-eye situation of the Romberg test, but lost balance after closing her eyes. The DVT was positive and the patient missed nine lines in the Snellen chart between visual acuity in the static situation and DVT. The DHI score was 84, representing profound disability and decreased quality of life. These results represent abnormal function in the horizontal semicircular canals, saccular and utricular system on both sides, loss of vestibular ocular reflex and a much-decreased quality of life.

The vestibular rehabilitation requires nine sessions (over 8 months), but the patient and rehabilitation team were well connected. The rehabilitation plan mainly focused on gaze stability and adaptation exercises including horizontal X1 exercise with near object, vertical X1 exercise with near object, horizontal X1 exercise with far object, vertical X1 exercise with far object, horizontal X1 exercise with near object and large target, vertical X1 exercise with near object and large target, diagonal gaze stability exercise, horizontal X2 exercise with near object, vertical X2 exercise with near object, horizontal head movement while waking with fixed vision, vertical head movement while waking with fixed vision and bouncing up and down a trampoline with fixed vision. We assessed the patient’s progress (quality of life, balance and oscillopsia) using DHI, the Romberg test and DVT through rehabilitation, as shown in (Table.1).

**Table 1:** Patient progress during vestibular rehabilitation program

	**Session**								
	**1**	**2**	**3**	**4**	**5**	**6**	**7**	**8**	**9**
DHI total score	84	88	82	72	68	54	38	16	14
Romberg test	+	+	+	+	+	+	-	-	-
DVT	+ (9 line)	+ (9 line)	+ (8 line)	+ (8 line)	+ (7 line)	+ (5 line)	+ (5 line)	-	-

These results showed no change in the first three sessions (roughly a 2-month period) in quality of life, vertigo, imbalance or oscillopsia. However, after this period, the patient showed gradual progress in all areas. The DHI score must change by at least 19 to be considered significant progress (12); therefore, change in this parameter from Session 1 to 4 is considered significant progress and clinically important. In last session, the DHI scores were close to normal and the Romberg test and DVT results were normal. These findings show complete compensation of the vestibular ocular reflex, normal balance function and a good quality of life.

## Discussion

Ototoxicity is the main cause of BVD (2,6,7) in developed countries. Aminoglycoside (especially gentamicin) still is a common medication for treating infectious disease resulting in severe vestibular damage. BVD represents an absence of positional nystagmus, bilateral low caloric response and observing catch-up saccade nystagmus in the HIT. In this patient, other tests (cVEMP & oVEMP) were also abnormal on both sides. These results show an extensive lesion that affects the whole vestibular system and, as mentioned by the patient, predominantly causing imbalance, falling and a low quality of life.

Although she was completely symptomatic in the first 2 months of treatment, the patient recovered completely at the end of nine sessions of vestibular rehabilitation. The motion ability of the patient, caused by surgical intervention kyphoscoliosis, was an additional challenge for vestibular rehabilitation. The patient is now able to walk without imbalance or falling down. She has normal physical and functional activities and has returned to school. The patient was fully supported by her family and performed all the exercises in regular and consistent way.

Considering the abnormal results in all vestibular assessments, substitution exercises would seem a logical approach for rehabilitation (13); however, considering her orthopedic problems and poor prognosis, this was not an optimal treatment in this case. The patient responded particularly well to the adaptation exercises, which may be related to her young age. 

Because of the uncommon nature of BVD, few articles regarding rehabilitation in these patients are available (8-11). These studies were performed on a low sample of patients that were much older, but showed recovery in some patients with BVD. Usually, the main course of treatment in these patients was substitution exercises. In one study, the authors found good recovery and improvement of dynamic visual acuity in 13 patients aged 47–73 years using adaptation and substitution exercises (11).

This case report shows a successful case of full recovery with adaptation exercises in a young patient with BVD who also suffered from an additional problem. This could suggest the possibility of using adaptation exercises in young patients with BVD; however this suggestion must first be verified with greater numbers of patients. BVD has a very adverse and profound effect on patients, but it is a rare condition, especially in younger ages. One main limitation is our inaccessibility to younger patients.

In general, vestibular rehabilitation is an effective way for treatment of patients with BVD. Dynamic visual acuity also could recover after vestibular rehabilitation (11,14).

## Conclusion

Vestibular rehabilitation could be an effective method for the treatment of complicated cases of BVD. Adaptation exercises may be useful in young patients with BVD.
